# Neurological Consequences of Sphingosine Phosphate Lyase Insufficiency

**DOI:** 10.3389/fncel.2022.938693

**Published:** 2022-09-14

**Authors:** Krishan B. Atreya, Julie D. Saba

**Affiliations:** ^1^National Centre for Biological Sciences, Tata Institute of Fundamental Research, Bangalore, India; ^2^Department of Pediatrics, School of Medicine, University of California, San Francisco, San Francisco, CA, United States

**Keywords:** sphingosine phosphate lyase, sphingosine-1-phosphate, sphingosine phosphate lyase insufficiency syndrome, peripheral neuropathy, brain development, NPHS14, *Drosophila*, axonopathy

## Abstract

In 2017, an inborn error of metabolism caused by recessive mutations in *SGPL1* was discovered. The disease features steroid-resistant nephrotic syndrome, adrenal insufficiency, and neurological defects. The latter can include sensorineural hearing loss, cranial nerve defects, peripheral neuropathy, abnormal brain development, seizures and/or neurodegeneration. *SGPL1* encodes the pyridoxal-5’-phosphate (PLP) dependent enzyme sphingosine phosphate lyase (SPL), and the condition is now referred to as SPL insufficiency syndrome (SPLIS). SPL catalyzes the final step in the degradative pathway of sphingolipids in which the bioactive sphingolipid sphingosine-1-phosphate (S1P) is irreversibly degraded to a long chain aldehyde and phosphoethanolamine (PE). SPL guards the only exit point for sphingolipid metabolism, and its inactivation leads to accumulation of various types of sphingolipids which have biophysical roles in plasma membrane rafts and myelin, and signaling roles in cell cycle progression, vesicular trafficking, cell migration, and programmed cell death. In addition, the products of the SPL reaction have biological functions including regulation of autophagic flux, which is important in axonal and neuronal integrity. In this review, the neurological manifestations of SPLIS will be described, and insights regarding the neurological consequences of SPL insufficiency from the study of brain-specific SPL knockout mice and *Drosophila* SPL mutants will be summarized.

## Introduction

**Figure 1:**
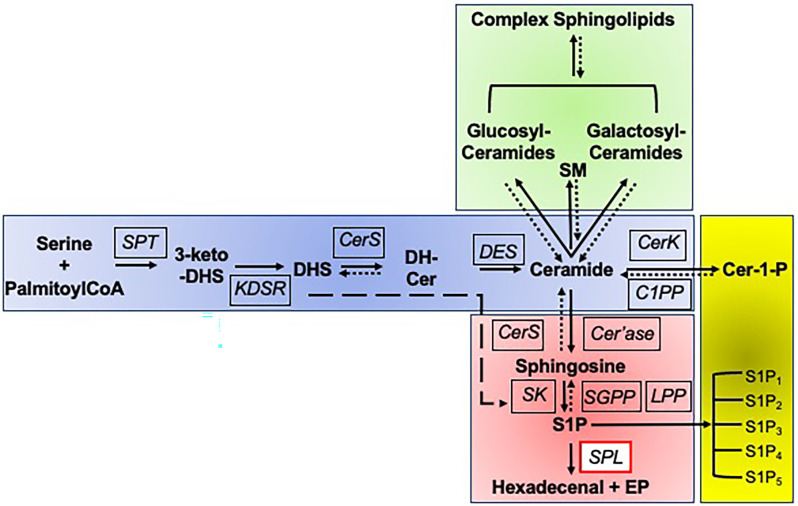
Sphingolipid biosynthetic and degradative pathways. Sphingolipid biosynthesis is initiated by the multi-subunit enzyme serine palmitoyltransferase (SPT) which catalyzes the condensation of serine and palymitoyl CoA to form 3-keto-dihydrosphingosine (3-keto-DHS). The reduction of 3-keto-DHS by 3-ketodihydrosphingosine reductase (KDSR) results in formation of the long chain amino base, dihydrosphingosine (DHS), which is subsequently acylated at its free amino group by one of five ceramide synthases (CerS), forming dihydroceramide (DH-Cer). The reversibility of this and subsequent steps is indicated by a dotted line and arrow. Alternatively, DHS can be directly phosphorylated to DHS1P by sphingosine kinases (SK) in the degradative pathway, as indicated by the long dashed line and arrow. The delta 4-desaturase (DES) encoded by DEGS1 then introduces a double bond into the DHS backbone, forming ceramide, the central building block of all sphingolipids. From ceramide, there are three possible directions, namely biosynthesis of sphingomyelin and glycosyl sphingolipids, phosphorylation, or degradation. Ceramide can be modified by addition of a phosphocholine group at the C1 position by sphingomyelin synthase, yielding sphingomyelin (SM). It can be glycosylated with either a glucose or galactose moiety to form glucosylceramides or galactosylceramides, followed by subsequent addition of sugars to form the complex array of glycolipids, the glycosylated sphingolipids. Each of these steps is reversible, thus routing higher order sphingolipids into the degradative pathway. Ceramide can be phosphorylated to the bioactive lipid ceramide-1-phosphate (Cer-1-P) by ceramide kinase (CerK), a step that can be reversed by ceramide phosphate phosphatase (C1PP). Alternatively, ceramide can enter the common degradative pathway that breaks down sphingolipids derived from *de novo* biosynthesis and from the recycling of higher order sphingolipids. The degradative pathway begins with ceramide being deacylated by ceramidase (Cer’ase), generating sphingosine. Sphingosine may be reutilized by CerS to regenerate ceramide in the salvage pathway, or it may continue through the degradative pathway, being phosphorylated by SK, generating the bioactive sphingolipid S1P. S1P can activate its five cognate G protein-coupled receptors (S1P1-S1P5). Sphingosine can be regenerated from S1P by S1P-specific phosphatases (SGPP) and nonspecific lipid phosphate phosphatases (LPP). Ultimately, all cellular S1P and DHS1P is irreversibly cleaved by sphingosine phosphate lyase (SPL), yielding two nonsphingolipid products, the long chain aldehyde hexadecenal (or hexadecanal, if DHS1P is the substrate) and phosphoethanolamine (PE).

Sphingolipids are a unique family of membrane lipids based on the core molecule, ceramide (Hannun and Obeid, 2018). Ceramide itself is comprised of a long chain base (LCB) anchor (sphingosine) in amide linkage with a fatty acid of variable chain length and saturation. Complex sphingolipids are formed by addition to the free hydroxyl group at the C1 position of ceramide of either phosphocholine by sphingomyelin synthase thereby producing sphingomyelin, or alternatively the addition of sequential sugar residues, giving rise to the diverse family of glycolipids known as glycosphingolipids (Figure 1).

Sphingolipids serve structural roles in plasma membranes by conferring biophysical characteristics and participating along with cholesterol in lipid rafts. In so doing, sphingolipids influence the activity and interactions of membrane proteins (Lingwood and Simons, 2010). Sphingolipids serve as receptors, blood group antigens, and regulators of cell-cell interactions. Sphingolipids also play important structural and signaling roles in the formation of membrane structures including cilia and microvilli (Kaiser et al., 2020). Further, they are enriched in the brain compared to other tissues and are key constituents of the myelin sheath. The metabolic recycling and degradation of complex sphingolipids—which takes place primarily in the lysosome—gives rise to bioactive intermediates such as ceramide, ceramide-1-phosphate and sphingosine-1-phosphate that participate in cell signaling events (Gomez-Larrauri et al., 2020).

The importance of sphingolipids to human neurobiology is illustrated most profoundly by the infantile onset neurodegeneration characteristic of sphingolipidoses, a family of inborn errors of metabolism caused by defects of lysosomal sphingolipid metabolism. Diseases such as Gaucher, Nieman Pick, Tay-Sachs, Sandhoff, Fabry, and GM1 gangliosidosis are examples of classic sphingolipidoses that exhibit neurological manifestations (Sandhoff, 2012). In fact, sphingolipidoses are among the earliest known inborn errors of metabolism to be identified, although the affected enzymes were not discovered until the 1960s and beyond. Lysosomal storage of sphingolipids is a pathological hallmark of sphingolipidoses. However, accumulation of sphingolipids in other organelles and cell membranes has been demonstrated in these lysosomal storage disorders, and the widespread dysregulation of membrane sphingolipids is suspected to contribute to the pathogenesis of the classical sphingolipidoses and their neurological complications.

In the last 30 years, yeast genetic strategies combined with elegant biochemical studies have elucidated the biochemical steps and nearly all the corresponding genes encoding the enzymes of sphingolipid metabolism as shown in Figure 1 (Dunn et al., 2019). Next generation sequencing of children with unexplained syndromes and presentations have revealed genetic conditions associated with mutations inactivating several of these recently discovered genes encoding non-lysosomal enzymes of sphingolipid metabolism. Like the classic sphingolipidoses, many of these atypical sphingolipidoses also have prominent neurological features including neurodegeneration and peripheral neuropathy. Sphingosine phosphate lyase insufficiency syndrome is one example of this novel class of “atypical” sphingolipid metabolic disorders.

## Sphingosine Phosphate Lyase Insufficiency Syndrome

Sphingosine phosphate lyase insufficiency syndrome (SPLIS), also known as NPHS14, is a recessive genetic disorder of sphingolipid metabolism that was discovered in 2017 (Lovric et al., 2017; Prasad et al., 2017). SPLIS is characterized by three main features: steroid-resistant nephrotic syndrome progressing rapidly to end stage renal disease, primary adrenal insufficiency manifesting mainly as glucocorticoid deficiency, and neurological defects involving the central and peripheral nervous systems (CNS, PNS; Weaver et al., 2020). Lymphopenia with varying degrees of immunodeficiency is a near universal feature of the condition, and ichthyosis or acanthosis of the skin, hypothyroidism, and retinal changes may be present. The condition may take a severe infantile form presenting as early as prenatally or in the first months of life with fetal hydrops, failure to thrive, seizures and rapid demise. In contrast, a milder form may present after infancy or beyond, manifesting initially as an axonal mononeuropathy that progresses slowly to involve other nerve groups, with the patient gradually acquiring other typical SPLIS features (Atkinson et al., 2017; Janecke et al., 2017; Settas et al., 2019). Less than 70 confirmed cases of SPLIS have been reported worldwide at this time. However, its true prevalence remains unknown. Many families of children diagnosed with SPLIS report the loss of other siblings due to fetal or perinatal demise, often in association with nonimmune hydrops or renal failure. In addition, older children affected by SPLIS often describe a prolonged diagnostic odyssey prior to finally receiving a genetic diagnosis, due to the current lack of familiarity of the condition by health care providers. Further, the incorporation of *SGPL1* into next generation sequencing diagnostic gene panels has been slow. Thus, many SPLIS cases on both the mild and severe ends of the clinical spectrum likely remain undiagnosed and unreported.

## Biochemistry and Genetics of SPLIS

SPLIS is caused by inheritance of bi-allelic pathogenic variants of *SGPL1*, the gene encoding sphingosine phosphate lyase (SPL). SPL is a pyridoxal-5’-phosphate dependent aldolase that catalyzes the ultimate step in the degradative pathway of sphingolipids (Kumar and Saba, 2009). In the reaction, the bioactive sphingolipid sphingosine-1-phosphate (S1P) is irreversibly degraded by a cleavage reaction at carbon C2–3, yielding a long chain aldehyde and phosphoethanolamine (PE). SPL guards the only exit point for sphingolipid metabolism, and its inactivation leads to accumulation of S1P and other LCB phosphates, as well as other sphingolipids which have structural roles in plasma membrane rafts, myelin, and signaling roles in cell cycle progression, vesicular trafficking, cell migration, and programmed cell death. In addition, the products of the SPL reaction have biological functions including the regulation of autophagic flux. Over 30 different mutations spanning the *SGPL1* coding and splice site regions have been observed in association with SPLIS, including missense mutations, nonsense mutations, splice site mutations, and one substitution at the cofactor-binding lysine (Saba et al., 2021). Although only a handful of pathogenic variants have been studied *in vitro*, those that have been evaluated were shown to inactivate or at least markedly reduce SPL enzyme function, in some cases reducing SPL protein abundance, and causing the accumulation of cellular S1P and other sphingolipids (Lovric et al., 2017). SPLIS patient plasma samples and skin-derived fibroblasts have shown higher levels of S1P and other sphingolipids than controls (Zhao et al., 2020). Pathogenic *SGPL1* mutations cluster in the central highly conserved cofactor-binding domain (Zhao et al., 2020). When the cofactor-binding domain is affected in both alleles, the risk of early death is higher than otherwise. These findings, together with *in vivo* gene rescue experiments described in the sections below, strongly suggest that the cause of the disease is SPL insufficiency.

## Neurological Manifestations of SPLIS

A comprehensive SPLIS natural history study has not yet been conducted, and detailed, systematically collected information regarding neurological involvement in SPLIS cases is lacking. Based on a study in 2020, we found the neurological features described in SPLIS patients and their prevalence included unspecified cranial nerve deficits, developmental delay, sensorineural hearing loss, strabismus, regression/progressive neurological deterioration, peripheral motor and sensory neuropathy, ptosis, microcephaly, and 1–2 descriptions of infantile hypotonia or spasticity (Table 1; Weaver et al., 2020). Retinal lesions have been documented rarely in SPLIS patients (Zhao et al., 2020). While seizures may be a part of the initial disease presentation, they often (although not always) appear to be related to adrenal insufficiency and resolve with hormone replacement therapy.

**Table 1:** Neurological deficits in SPLIS patients.*

**Neurological deficit**	**Percentage of SPLIS patients affected**
Cranial nerve deficits	24
Developmental delay	20
Sensorineural hearing loss	18
Seizures	15
Strabismus	13
Regression/progressive neurological deterioration	13
Peripheral motor and sensory neuropathy	11
Microcephaly	10
Infantile hypotonia or spasticity	8
Ptosis	4
Retinopathy	several cases
Major brain developmental abnormality	a single case

*From Weaver et al. (2020).

Abnormal results of brain imaging have been reported in a handful of patients with SPLIS. A systematic review of brain imaging results from 15 SPLIS cases was performed including 10 cases reported in the literature and five cases (four new and one previously reported) wherein detailed magnetic resonance imaging (MRI) results had been obtained (Martin et al., 2020). Recurring features in SPLIS evident on brain MRI included isolated callosal agenesis or dysgenesis and involvement of the deep gray nuclei including globus pallidus, thalamus, caudate, putamen, brainstem, cerebellum and dentate nucleus. Atrophy, when present, was often progressive, and brain lesions also worsened over time. In one older patient, multiple focal lesions in the cerebral and cerebellar cortex and subcortical white matter developed over time. The findings could be consistent with a variety of pathogenic mechanisms of toxic, metabolic, mitochondrial, infectious, and postinfectious origin.

In several SPLIS patients diagnosed in infancy, the dopaminergic pathways appear to be injured, based on progressive lesions observed especially on T2 weighted images of the globus pallidus, dentate nucleus, red nucleus and substantia nigra on successive brain MRI images (Martin et al., 2020). These pathways are known to exhibit high metabolic rates as well as high levels of mineral transport (Nguyen et al., 2019). Sphingolipid accumulation could lead to changes in mitochondrial function, energy production, myelin composition, blood supply and vesicular trafficking—all of which could be particularly injurious to the neurons of dopaminergic pathways, which have selective vulnerabilities to these types of stress (Nguyen et al., 2019). In fact, both ceramide and S1P have been shown to cause neurotoxicity in cultured neurons (Milstien et al., 2007; Hagen et al., 2009).

Additional clues suggest the possibility that lesions reflect changes in calcium or iron deposition, as they appear similar to the neurodegeneration and iron deposition lesions observed in patients with pantothenate kinase-associated neurodegeneration, although these have not been confirmed (Klopstock et al., 2019). No evidence of demyelination was present. In contrast to the findings on brain MRI, computed tomography scans did not reveal abnormalities in SPLIS patients even when MRI results were abnormal at the same time point for the same patient.

In addition to callosal dysgenesis noted in several patients with SPLIS, one case manifesting as abnormal brain development was reported by Bamborschke et al. (2018). In this unusual report, a newborn with multisystem involvement was found by brain MRI to exhibit a simplified brain gyral pattern, hypoplastic temporal lobes, cerebellar hypoplasia, generalized cortical atrophy and hyperintense lesions in the pons. Thus far, this is the only reported case of a severe congenital brain developmental anomaly in SPLIS.

## Neurological Consequences of SPL Insufficiency in Mouse Models

**Figure 2:**
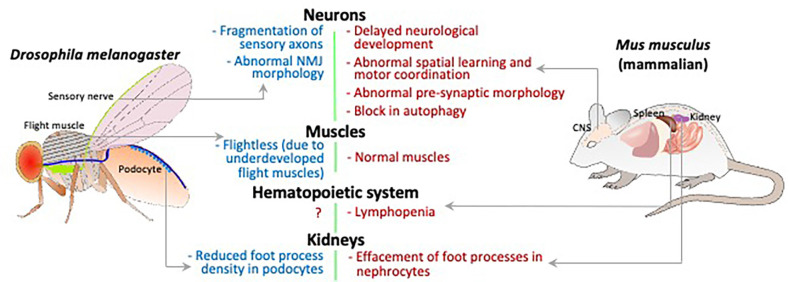
Schematic comparing the *Drosophila* and mammalian organ systems for phenotypes caused by loss of S1P lyase activity. The relevant organs/tissue are labeled and phenotypes indicated with gray arrows.

Global *Sgpl1* knockout (KO) mice are born at normal frequency but only survive for a few weeks (Schmahl et al., 2007). Despite a lack of obvious neurological phenotypes or brain pathology in the KO mice, careful analysis of their early neurological development revealed reduced grip strength and delayed achievement of milestones including eye opening, hearing, and adult pattern walking in comparison to wild type littermates (Figure 2; Zhao et al., 2021). Adeno-associated virus 9-mediated *SGPL1* gene rescue administered to *Sgpl1* KO pups in their first days of life prevented their neurodevelopmental delay (Zhao et al., 2021).

Van Echten-Deckert and colleagues had shown previously that S1P is toxic to neurons, disrupts calcium homeostasis, and promotes endoplasmic reticulum stress (Hagen et al., 2011; Karaca et al., 2014). To explore the neurological consequences of SPL insufficiency *in vivo*, they generated a brain-specific SPL knockout mouse (SPL^BrainKO^) that lives to adulthood by inducing *Sgpl1* disruption in brain *via* expression of a Cre transgene under the control of the nestin promoter in a *Sgpl1* floxed background (Mitroi et al., 2016). SPL was markedly reduced and tissue S1P levels were high in the brain of the SPL^BrainKO^ mice. SPL^BrainKO^ mice exhibited abnormalities of motor and behavioral function including memory, spatial learning and motor coordination (Figure 2). SPL^BrainKO^ mice brain histology revealed a subtle reduction in thickness of the dentate gyrus, and ultrastructural analysis of hippocampal excitatory neurons coupled with electrophysiology measurements revealed changes in pre-synaptic morphology accompanied by a reduction in short term plasticity. The investigators found that several pre-synaptic proteins were markedly reduced in SPL^BrainKO^ compared to WT brains and cultured neurons. The observed changes were attributed to upregulation of protein ubiquitination and proteasomal activity in the KO brains. Normal synaptic physiology and the levels of presynaptic protein markers were restored by inhibition of the proteasome (Mitroi et al., 2016). Additional findings revealed by the SPL^BrainKO^ model suggest that SPL and S1P are regulators of the unfolded protein response that may be triggered in neurons by changes in calcium and can lead to disruption of normal presynaptic architecture and physiology with consequences in motor and behavioral function. Interestingly, when the authors of the study generated a second mouse model in which SPL deficiency occurs postnatally and in a forebrain-restricted pattern, they observed no accumulation of S1P in the KO brain, and no manifestation of the phenotypes found in the SPL^BrainKO^. This may be explained by residual SPL activity in the brain tissue sufficient to maintain S1P metabolism, in contrast to the SPL^BrainKO^, in which *Sgpl1* disruption occurs in neuronal progenitor cells before birth and affects all regions of the brain.

Further study of the SPL^BrainKO^ mice brain and hippocampal neurons grown in culture revealed a block in neuronal autophagic flux in the SPL-deficient state (Figure 2; Mitroi et al., 2017). This was attributed at least in part to a reduction in the SPL product PE, whose incorporation into the phospholipid phosphatidylethanolamine (PtdE) is needed for lipidation of the protein LC3, which itself contributes to maturation of the autophagosome (Rockenfeller et al., 2015; Tommasino et al., 2015). Defective autophagy in the SPL^BrainKO^ hippocampal neurons was associated with abnormally high levels of aggregation-prone proteins including synuclein and amyloid precursor protein (APP) in KO brain tissues (Mitroi et al., 2017).

The features of the SPL^BrainKO^ brain, including defective autophagy, enhanced proteasomal activity, and accumulation of aggregation-prone proteins such as APP and alpha-synuclein are important in the pathophysiology of neurodegenerative diseases such as Parkinson’s Disease and Alzheimer’s Disease (Suh and Checler, 2002). With the recognition that some children affected by SPLIS exhibited developmental regression accompanied by signs of brain atrophy and lesions of the basal ganglia detected on brain magnetic resonance images, the group of van Echten-Deckert undertook a more detailed investigation of the SPL^BrainKO^ model to look for signs of pathology associated with neurodegenerative diseases. They found the hippocampus and cortex of the KO mouse brain exhibited increased phosphorylation of tau, a microtubule-associated protein that contributes to intraneuronal neurofibrillary tangles in Alzheimer disease (Alam et al., 2020). They also found the KO brain tissues to be higher in acetylated forms of histone 3 and 4, consistent with the deregulation of histone acetylation observed in Alzheimer Disease and implicated in memory loss. Both of the observed changes appeared to be mediated by disruption of calcium homeostasis, as they could be reversed by calcium chelation (Alam et al., 2020). Thus, the generation and detailed characterization of the SPL^BrainKO^ mouse model has provided substantial insight into the molecular mechanism of neuropathology in SPLIS.

## Neurological Defects in A *Drosophila* Model of SPLIS

The fruitfly *Drosophila melanogaster* has served as a very useful model of lysosomal storage disorders, including sphingolipidoses (Rigon et al., 2021). The high degree of conservation seen across animal taxa in genes that regulate lysosome biogenesis and function, allows *Drosophila* mutants to phenocopy many of the features seen in human patients with sphingolipidoses. The strength of the fly model of SPLIS lies in the unparalleled ease with which genetic analyses can be conducted. Further, clonal analyses or tissue-specific and temporally regulated changes in gene expression can help unravel the cell autonomous vs. non-cell autonomous contribution of S1P lyase deficiency.

In *Drosophila*, the gene that codes for S1P lyase, *sply*, has one reported loss of function allele, *sply^05091^*, produced by the insertion of a transposable element within the locus. Northern analysis and *in-situ* hybridization suggest that this is a null allele, thought this has not yet been confirmed by protein analysis. Mutant flies have increased levels of LCBs and LCB phosphates, and improperly developed flight muscles (Figure 2; Herr et al., 2003). The adults rapidly become sterile due to induction of apoptosis in the ovaries and testes (Phan et al., 2007). Crucially, there are many non-neuronal defects in *sply* mutants that mimic features of SPLIS, the most striking of which is disruption of podocyte function. Both mouse and *Drosophila* podocytes that are deficient in S1P lyase display defective foot-processes, and the fly podocytes also shows reduced uptake of albumin, indicative of disrupted function (Lovric et al., 2017). Thus, it is appropriate to try to explore the *sply* mutant to investigate the neurological manifestations of SPLIS.

Analysis of the giant fiber neuromuscular pathway in the *sply^05091^* mutant flies showed no electrophysiological defects (Herr et al., 2003), but elsewhere, induction of RNAi against *sply* in neurons resulted in morphological defects in the larval neuromuscular junction, and fragmentation of sensory neuronal axons in wing margins (Figure 2; Atkinson et al., 2017). These differences might be explained by the fact that synaptic homeostatic mechanisms can maintain synaptic function, even while changes occur in neuronal and synaptic morphology (Goel and Dickman, 2021).

Interestingly, the two molecules whose levels are most strongly impacted by S1P lyase activity, S1P and PE, have been implicated in neuronal or synaptic function in *Drosophila*. Overexpression of SK2 in *Drosophila* photoreceptors, which leads to an increase in levels of S1P and dihydro-S1P (and presumably also increases production of the S1P lyase products), causes degeneration of photoreceptor neurons by promoting rapid endocytosis of rhodopsin and the TRP cation channel (Yonamine et al., 2011). Similarly, in a fruitfly model of fragile X-associated tremor/ataxia syndrome, wherein overexpression of a CGG repeat leads to degeneration of the eyes, reducing the expression of SK2 exacerbates the phenotype (Kong et al., 2019). PE produced as a result of S1P lyase activity has been implicated in the production of PtdE to regulate autophagy in neurons (Mitroi et al., 2017). Given that axons and dendrites are particularly vulnerable to defects in autophagy (Yang et al., 2013), further experiments in *Drosophila sply* mutants could reveal neuronal dysfunction as seen in SPLIS patients. However, PtdE itself has other roles in neurons. In a screen for defects in *Drosophila* photoreceptors, mutations in components of the PtdE synthesis pathway led to loss of synaptic vesicles and degeneration of photoreceptor neurons (Tsai et al., 2019). This was attributed to an increase in the activity of the transcription factor SREBP, which is regulated by PtdE in contrast to the cholesterol-dependent regulation of SREBP in humans (Dobrosotskaya et al., 2002). The same mechanism is also required for the regulation of dendritic arborization in *Drosophila* sensory neurons (Meltzer et al., 2017). Given these neuronal roles for S1P and PE, it is quite probable that S1P lyase activity is required for the regulation of synaptic activity and neuronal morphology.

## Summary

In summary, SPLIS is an atypical sphingolipidosis that can manifest as a peripheral neuropathy involving axonal degeneration and/or a progressive neurodegenerative condition, with no evidence of demyelination in either CNS or PNS involvement. Vertebrate and invertebrate models of SPL insufficiency have revealed disturbances of key cellular functions critical for neuronal health, including autophagic flux, proteasome activity and removal of aggregation-prone proteins, calcium homeostasis, unfolded protein response, and vesicular trafficking. These findings are reminiscent of changes associated with the classical sphingolipidoses (Eckhardt, 2010). Thus, the study of SPLIS and other atypical sphingolipidoses is beginning to reveal common molecular and cellular features that underly associated neuropathology and may help to elucidate the fundamental role sphingolipids play in neuronal development and function.

## Contribution to The Field

Sphingolipids are a unique family of lipids that confer biophysical characteristics to plasma membranes, contribute to the composition and stability of myelin, and can be degraded to bioactive intermediates with cell signaling functions. The classical sphingolipidoses are storage diseases caused by inactivation of lysosomal enzymes required for degrading glycosphingolipids and sphingomyelin (Schuette et al., 1999). Many sphingolipidoses present with early onset neurodegeneration caused by demyelination and/or dysregulated autophagy (Arenz, 2017). Some gene variants of sphingolipid degrading enzymes are linked to sporadic Parkinson’s Disease. SPLIS is a newly recognized disorder of sphingolipid metabolism that can involve defects of the central and peripheral nervous system. SPLIS represents an emerging class of atypical sphingolipidoses that do not involve lysosomal enzymes of sphingolipid metabolism. Despite this distinction, atypical sphingolipid disorders often manifest with neurological impairment as a key clinical feature. Further, evidence from murine knockout models of SPLIS suggest that common mechanisms of disease pathogenesis involving autophagy and vesicular trafficking defects may explain the neurological involvement characteristic of both classical and atypical sphingolipid metabolic disorders. Thus, although SPLIS is a rare condition, study of vertebrate and invertebrate models of SPL insufficiency may provide insights into the fundamental role sphingolipids play in neuronal development and function.

## Author Contributions

JS and KA wrote, edited, and approved the manuscript in final form. All authors contributed to the article and approved the submitted version.

## Funding

We are grateful for the support from the California Institute of Regenerative Medicine DISC2-13072, NIH Public Health Grant DK115669, Swim Across America Foundation, and UCSF Innovation Catalyst Program (JS) for this review.

## Abbreviations

APP: Amyloid precursor protein
CNS: central nervous system
KO: knockout
LCB: long chain base
LCBP: long chain base phosphate
MRI: magnetic resonance imaging
PE: phosphoethanolamine
PLP: pyridoxal-5’-phosphate
PNS: peripheral nervous system
PtdE: phosphatidylethanolamine
S1P: sphingosine-1-phosphate
SK2: sphingosine kinase 2
SPL: sphingosine phosphate lyase
SPLIS: sphingosine phosphate lyase insufficiency syndrome.

## References

[B1] Alam S. Piazzesi A. Abd El Fatah M. Raucamp M. Van Echten-Deckert G. (2020). Neurodegeneration caused by S1P-lyase deficiency involves calcium-dependent tau pathology and abnormal histone acetylation. Cells 9:2189. 10.3390/cells9102189 32998447 PMC7599816

[B2] Arenz C. (2017). Recent advances and novel treatments for sphingolipidoses. Future Med. Chem. 9, 1687–1700. 10.4155/fmc-2017-0065 28857617

[B3] Atkinson D. Nikodinovic Glumac J. Asselbergh B. Ermanoska B. Blocquel D. Steiner R.. (2017). Sphingosine 1-phosphate lyase deficiency causes charcot-marie-tooth neuropathy. Neurology 88, 533–542. 10.1212/WNL.0000000000003595 28077491 PMC5304460

[B4] Bamborschke D. Pergande M. Becker K. Koerber F. Dotsch J. Vierzig A.. (2018). A novel mutation in sphingosine-1-phosphate lyase causing congenital brain malformation. Brain Dev. 40, 480–483. 10.1016/j.braindev.2018.02.008 29501407

[B5] Dobrosotskaya I. Seegmiller A. Brown M. Goldstein J. Rawson R. (2002). Regulation of SREBP processing and membrane lipid production by phospholipids in *Drosophila*. Science 296, 879–883. 10.1126/science.1071124 11988566

[B6] Dunn T. M. Tifft C. J. Proia R. L. (2019). A perilous path: the inborn errors of sphingolipid metabolism. J. Lipid Res. 60, 475–483. 10.1194/jlr.S091827 30683667 PMC6399501

[B7] Eckhardt M. (2010). Pathology and current treatment of neurodegenerative sphingolipidoses. Neuromolecular Med. 12, 362–382. 10.1007/s12017-010-8133-7 20730629

[B8] Goel P. Dickman D. (2021). Synaptic homeostats: latent plasticity revealed at the Drosophila neuromuscular junction. Cell. Mol. Life Sci. 78, 3159–3179. 10.1007/s00018-020-03732-3 33449150 PMC8044042

[B9] Gomez-Larrauri A. Presa N. Dominguez-Herrera A. Ouro A. Trueba M. Gomez-Munoz A. (2020). Role of bioactive sphingolipids in physiology and pathology. Essays Biochem. 64, 579–589. 10.1042/EBC20190091 32579188

[B10] Hagen N. Hans M. Hartmann D. Swandulla D. Van Echten-Deckert G. (2011). Sphingosine-1-phosphate links glycosphingolipid metabolism to neurodegeneration via a calpain-mediated mechanism. Cell Death Differ. 18, 1356–1365. 10.1038/cdd.2011.7 21331079 PMC3172106

[B100] Hagen N. Van Veldhoven P. P. Proia R. L. Park H. Merrill A. H. Jr. Van Echten-Deckert G. (2009). Subcellular origin of sphingosine 1-phosphate is essential for its toxic effect in lyase-deficient neurons. J. Biol. Chem. 284, 11346–11353. 10.1074/jbc.M807336200 19251691 PMC2670140

[B11] Hannun Y. A. Obeid L. M. (2018). Sphingolipids and their metabolism in physiology and disease. Nat. Rev. Mol. Cell Biol. 19, 175–191. 10.1038/nrm.2017.107 29165427 PMC5902181

[B12] Herr D. R. Fyrst H. Phan V. Heinecke K. Georges R. Harris G. L.. (2003). Sply regulation of sphingolipid signaling molecules is essential for *Drosophila* development. Development 130, 2443–2453. 10.1242/dev.00456 12702658

[B13] Janecke A. R. Xu R. Steichen-Gersdorf E. Waldegger S. Entenmann A. Giner T.. (2017). Deficiency of the sphingosine-1-phosphate lyase SGPL1 is associated with congenital nephrotic syndrome and congenital adrenal calcifications. Hum. Mutat. 38, 365–372. 10.1002/humu.23192 28181337 PMC5384969

[B14] Kaiser F. Huebecker M. Wachten D. (2020). Sphingolipids controlling ciliary and microvillar function. FEBS Lett. 594, 3652–3667. 10.1002/1873-3468.13816 32415987

[B15] Karaca I. Tamboli I. Y. Glebov K. Richter J. Fell L. H. Grimm M. O.. (2014). Deficiency of sphingosine-1-phosphate lyase impairs lysosomal metabolism of the amyloid precursor protein. J. Biol. Chem. 289, 16761–16772. 10.1074/jbc.M113.535500 24808180 PMC4059120

[B16] Klopstock T. Tricta F. Neumayr L. Karin I. Zorzi G. Fradette C.. (2019). Safety and efficacy of deferiprone for pantothenate kinase-associated neurodegeneration: a randomised, double-blind, controlled trial and an open-label extension study. Lancet Neurol. 18, 631–642. 10.1016/S1474-4422(19)30142-5 31202468

[B17] Kong H. E. Lim J. Zhang F. Huang L. Gu Y. Nelson D. L.. (2019). Metabolic pathways modulate the neuronal toxicity associated with fragile X-associated tremor/ataxia syndrome. Hum. Mol. Genet. 28, 980–991. 10.1093/hmg/ddy410 30476102 PMC6400045

[B18] Kumar A. Saba J. D. (2009). Lyase to live by: sphingosine phosphate lyase as a therapeutic target. Expert Opin. Ther. Targets 13, 1013–1025. 10.1517/14728220903039722 19534571 PMC2774446

[B19] Lingwood D. Simons K. (2010). Lipid rafts as a membrane-organizing principle. Science 327, 46–50. 10.1126/science.1174621 20044567

[B20] Lovric S. Goncalves S. Gee H. Y. Oskouian B. Srinivas H. Choi W. I.. (2017). Mutations in sphingosine-1-phosphate lyase cause nephrosis with ichthyosis and adrenal insufficiency. J. Clin. Invest. 127, 912–928. 10.1172/JCI89626 28165339 PMC5330730

[B21] Martin K. W. Weaver N. Alhasan K. Gumus E. Sullivan B. R. Zenker M.. (2020). MRI spectrum of brain involvement in sphingosine-1-phosphate lyase insufficiency syndrome. Am. J. Neuroradiol. 41, 1943–1948. 10.3174/ajnr.A6746 32855188 PMC7661081

[B22] Meltzer S. Bagley J. A. Perez G. L. O’brien C. E. Devault L. Guo Y.. (2017). Phospholipid homeostasis regulates dendrite morphogenesis in Drosophila sensory neurons. Cell Rep. 21, 859–866. 10.1016/j.celrep.2017.09.089 29069593 PMC5687885

[B23] Milstien S. Gude D. Spiegel S. (2007). Sphingosine-1-phosphate in neural signalling and function. Acta Paediatr. 96, 40–43. 10.1111/j.1651-2227.2007.00206.x 17391440

[B24] Mitroi D. N. Deutschmann A. U. Raucamp M. Karunakaran I. Glebov K. Hans M.. (2016). Sphingosine 1-phosphate lyase ablation disrupts presynaptic architecture and function via an ubiquitin- proteasome mediated mechanism. Sci. Rep. 6:37064. 10.1038/srep37064 27883090 PMC5121647

[B25] Mitroi D. N. Karunakaran I. Graler M. Saba J. D. Ehninger D. Ledesma M. D.. (2017). SGPL1 (sphingosine phosphate lyase 1) modulates neuronal autophagy via phosphatidylethanolamine production. Autophagy 13, 885–899. 10.1080/15548627.2017.1291471 28521611 PMC5446076

[B26] Nguyen M. Wong Y. C. Ysselstein D. Severino A. Krainc D. (2019). Synaptic, mitochondrial and lysosomal dysfunction in Parkinson’s disease. Trends Neurosci. 42, 140–149. 10.1016/j.tins.2018.11.001 30509690 PMC6452863

[B27] Phan V. H. Herr D. R. Panton D. Fyrst H. Saba J. D. Harris G. L. (2007). Disruption of sphingolipid metabolism elicits apoptosis-associated reproductive defects in *Drosophila*. Dev. Biol. 309, 329–341. 10.1016/j.ydbio.2007.07.021 17706961 PMC2094363

[B28] Prasad R. Hadjidemetriou I. Maharaj A. Meimaridou E. Buonocore F. Saleem M.. (2017). Sphingosine-1-phosphate lyase mutations cause primary adrenal insufficiency and steroid-resistant nephrotic syndrome. J. Clin. Invest. 127, 942–953. 10.1172/JCI90171 28165343 PMC5330744

[B29] Rigon L. De Filippis C. Napoli B. Tomanin R. Orso G. (2021). Exploiting the potential of *Drosophila* models in lysosomal storage disorders: pathological mechanisms and drug discovery. Biomedicines 9:268. 10.3390/biomedicines9030268 33800050 PMC8000850

[B30] Rockenfeller P. Koska M. Pietrocola F. Minois N. Knittelfelder O. Sica V.. (2015). Phosphatidylethanolamine positively regulates autophagy and longevity. Cell Death Differ. 22, 499–508. 10.1038/cdd.2014.219 25571976 PMC4326582

[B31] Saba J. D. Keller N. Wang J. Y. Tang F. Slavin A. Shen Y. (2021). Genotype/phenotype interactions and first steps toward targeted therapy for sphingosine phosphate lyase insufficiency syndrome. Cell Biochem. Biophys. 79, 547–559. 10.1007/s12013-021-01013-9 34133011

[B32] Sandhoff K. (2012). My journey into the world of sphingolipids and sphingolipidoses. Proc. Jpn. Acad. Ser. B Phys. Biol. Sci. 88, 554–582. 10.2183/pjab.88.554 23229750 PMC3552047

[B33] Schmahl J. Raymond C. S. Soriano P. (2007). PDGF signaling specificity is mediated through multiple immediate early genes. Nat. Genet. 39, 52–60. 10.1038/ng1922 17143286

[B34] Schuette C. G. Doering T. Kolter T. Sandhoff K. (1999). The glycosphingolipidoses-from disease to basic principles of metabolism. Biol. Chem. 380, 759–766. 10.1515/BC.1999.096 10494825

[B35] Settas N. Persky R. Faucz F. R. Sheanon N. Voutetakis A. Lodish M.. (2019). SGPL1 deficiency: a rare cause of primary adrenal insufficiency. J. Clin. Endocrinol. Metab. 104, 1484–1490. 10.1210/jc.2018-02238 30517686 PMC6435096

[B36] Suh Y. H. Checler F. (2002). Amyloid precursor protein, presenilins and alpha-synuclein: molecular pathogenesis and pharmacological applications in Alzheimer’s disease. Pharmacol. Rev. 54, 469–525. 10.1124/pr.54.3.469 12223532

[B37] Tommasino C. Marconi M. Ciarlo L. Matarrese P. Malorni W. (2015). Autophagic flux and autophagosome morphogenesis require the participation of sphingolipids. Apoptosis 20, 645–657. 10.1007/s10495-015-1102-8 25697338

[B38] Tsai J. W. Kostyleva R. Chen P. L. Rivas-Serna I. M. Clandinin M. T. Meinertzhagen I. A.. (2019). Transcriptional feedback links lipid synthesis to synaptic vesicle pools in *Drosophila* photoreceptors. Neuron 101, 721–737.e4. 10.1016/j.neuron.2019.01.015 30737130 PMC8053036

[B39] Weaver K. N. Sullivan B. Hildebrandt F. Strober J. Cooper M. Prasad R.. (2020). “Sphingosine phosphate lyase insufficiency syndrome,” in GeneReviews, eds Adam M. P. Ardinger H. H. Pagon R. A. Wallace S. E. Bean L. J. H. Stephens K. Amemiya A. (Seattle, WA: University of Washington). 33074640

[B40] Yang Y. Coleman M. Zhang L. Zheng X. Yue Z. (2013). Autophagy in axonal and dendritic degeneration. Trends Neurosci. 36, 418–428. 10.1016/j.tins.2013.04.001 23639383 PMC3787524

[B41] Yonamine I. Bamba T. Nirala N. K. Jesmin N. Kosakowska-Cholody T. Nagashima K.. (2011). Sphingosine kinases and their metabolites modulate endolysosomal trafficking in photoreceptors. J. Cell Biol. 192, 557–567. 10.1083/jcb.201004098 21321100 PMC3044112

[B42] Zhao P. Liu I. D. Hodgin J. B. Benke P. I. Selva J. Torta F.. (2020). Responsiveness of sphingosine phosphate lyase insufficiency syndrome to vitamin B6 cofactor supplementation. J. Inherit. Metab. Dis. 43, 1131–1142. 10.1002/jimd.12238 32233035 PMC8072405

[B43] Zhao P. Tassew G. B. Lee J. Y. Oskouian B. Munoz D. P. Hodgin J. B.. (2021). Efficacy of AAV9-mediated SGPL1 gene transfer in a mouse model of S1P lyase insufficiency syndrome. JCI Insight 6:e145936. 10.1172/jci.insight.145936 33755599 PMC8119223

